# Comparison of post-authorisation measures from regulatory authorities with additional evidence requirements from the HTA body in Germany – are additional data requirements by the Federal Joint Committee justified?

**DOI:** 10.1186/s13561-016-0124-4

**Published:** 2016-09-29

**Authors:** Jörg Ruof, Thomas Staab, Charalabos-Markos Dintsios, Jakob Schröter, Friedrich Wilhelm Schwartz

**Affiliations:** 1 Roche Pharma AG, Emil-Barrell-Str. 1, 79639 Grenzach-Wyhlen, Germany; 2 Medical School of Hanover, Hanover, Germany; 3 Health Services Research and Health Economics, Heinrich Heine University, Düsseldorf, Germany; 4 Baden-Württemberg Cooperative State University, Lörrach, Germany

**Keywords:** Marketing authorisation, Post-authorisation measure, (Early) benefit assessment, Conditional appraisal, EMA, G-BA

## Abstract

**Objectives:**

The aim of this study was to compare post-authorisation measures (PAMs) from the European Medicines Agency (EMA) with data requests in fixed-termed conditional appraisals of early benefit assessments from the German Federal Joint Committee (G-BA).

**Methods:**

Medicinal products with completed benefit assessments during an assessment period of 3.5 years were considered. PAMs extracted from European Public Assessment Reports (EPARs) were compared with data requests issued by the G-BA in the context of conditional appraisals.

**Results:**

Twenty conditional appraisals (19 products) and 34 EPARs containing PAMs (33 products) were identified. Data categories (efficacy, safety, etc.), data types (type of study required to address the request) and clarity of requests were determined. Conditional appraisals disproportionately focused on oncology products (13/19 products with conditional appraisals vs. 14/33 products with PAMs). No clear rationale for the G-BA issuing conditional appraisals could be identified in public sources. Both EMA and G-BA requested mainly efficacy and safety data (44/54 and 23/35 categories requested, respectively); however, 28/35 G-BA data requirements went beyond requests made by the EMA. Almost half of the G-BA requests (9/20), but no PAMs, were unclear, and no methodological guidance for fulfilling the data requirements was provided by the G-BA.

**Conclusions:**

Better alignment between data requests from regulatory authorities and health technology assessment bodies is strongly recommended.

## Background

At the time of marketing authorisation, the available information relating to a medicine may not yet be sufficient to fully assess the benefit/risk profile to the desired degree of certainty or may lack aspects of interests to the Committee for Medicinal Products for Human Use (CHMP) that are requested to be provided after marketing authorisation is granted. Therefore, marketing authorisation agencies such as the U.S. Food and Drug Administration (FDA) or the European Medicines Agency (EMA) may require the generation of additional data, e.g. in the form of clinical studies.

The FDA refers to these additional requirements as post-marketing requirements and commitments. These gather additional information about a product's safety, efficacy or optimal use [1]. The EMA can require post-authorisation measures (PAMs) if the Agency’s committees consider the generation of additional data necessary from a “public health perspective to complement the available data with additional data about the safety and, in certain cases, the efficacy of authorised medicinal products”. Thus, like their FDA equivalent, PAMs are mainly required in order to answer open questions regarding the risk/benefit profile or safety concerns of a product [2].

The European Public Assessment Report (EPAR) provides details of the measures required for each product and the agreed time frame for completion. PAMs are a well-established component of the centralised procedure for marketing authorisation in Europe and manufacturers are able to draw upon a wealth of guidance. Generally, each PAM is discussed in detail by the EMA and the manufacturer to define the clinical question and the trial design, including details of the patient population, intervention, comparison and outcomes of proposed studies [2]. PAMs are especially imposed on conditional marketing authorisations or when marketing authorisations have been granted under exceptional circumstances.

Since 2011, following the introduction of the Act for Restructuring the Pharmaceutical Market in Statutory Health Insurance (*Arzneimittel – Markt – Neu – Ordnungs – Gesetz*, AMNOG), a product has to undergo an early benefit assessment after marketing authorisation and launch in Germany. The assessment aims to determine the additional benefit of a product against a pre-defined appropriate comparator therapy in terms of morbidity, mortality, quality of life and safety. The extent of additional benefit granted will in turn influence the reimbursement price. Scientific information is presented by the manufacturer in the form of a benefit dossier according to a formalistic template and guidelines from the Federal Joint Committee (*Gemeinsamer Bundesausschuss*, G-BA). In general, the benefit assessment is conducted as a two-step approach encompassing i) scientific assessment by the Institute for Quality and Efficiency in Health Care (IQWiG) and ii) appraisal by the G-BA; for orphan drugs, both, assessment and appraisal, are performed by the G-BA. A decision is made on the extent of the additional benefit (major, considerable, minor, no benefit or benefit less than comparator) and the level of evidence (proof, indication, hint) [3–5]. Details of the early benefit assessment procedure have been described previously [6, 7].

Based on German social law, the G-BA has the option to issue conditional appraisals [3, 5]. This is usually done if no reliable conclusions regarding the extent of additional benefit can be drawn from the submitted data [8]. After a pre-specified time frame, the G-BA will review the additionally submitted scientific data and subsequently re-assess a product’s additional benefit in a new procedure. The G-BA’s decision rationale (*Tragende Gründe*) provides further information on the requested data and the time frame for submission of the updated dossier containing the additional data [8]. In contrast to PAMs, experience regarding conditional appraisals and additional data requests by the G-BA is currently limited to a 5-year time frame [8].

In this study, we compared the requests for additional data by the EMA (PAMs) and the G-BA (conditional appraisals) to illustrate differences in the categories and types of requested data and to identify differences between PAMs and conditional appraisals in the still rather new and developing early benefit assessment process in Germany.

## Methods

A step-wise approach was used for this analysis, comprising identification of all early benefit assessments of new medicines or new indications during the first 3.5 years after the introduction of the procedure in Germany, identification of the corresponding EPARs/EPAR variations and subsequent classification of data requests in conditional appraisals/PAMs. Parameters analysed included data categories, data types and clarity of data requests.

### Identification and inclusion of benefit assessments

Benefit assessments conducted from 1 January 2011 (the date of coming into effect of the new legislation) to 1 July 2014, excluding assessments of currently marketed pharmaceuticals, and the respective appraisals were retrieved from the G-BA website [8]. Conditional appraisals were identified and the requested data were extracted from the G-BA decision rationale [8]. For re-assessments of products for the same indication following additional data being filed by the manufacturer, only the latest assessment was included.

### Identification and inclusion of EPARs and PAMs

PAMs and information on conditional approval or approval under exceptional circumstances for the corresponding products were obtained from the EPARs available on the EMA website [9]. For each product, ongoing and fulfilled PAMs were extracted from Annex II of the EPAR and from the Procedural Steps, respectively. The latest version of each document as of August 2014 was used for each product. Products granted marketing authorisation for an additional indication were counted twice, as an EPAR variation report is available for the new indication and PAMs can be clearly distinguished between the two indications. Products not undergoing the centralised procedure were excluded from the EPAR analysis as no EPAR was available.

### Classification of therapeutic indication

Therapeutic indications of products with conditional appraisals or PAMs were classified as cardiovascular disorders, infectious diseases, metabolic disorders, neurological disorders, oncology, ophthalmology, respiratory disorders or others, according to the information on the G-BA website [8].

### Quality of data from RCTs for oncology drugs with conditional vs. unconditional appraisals

For randomised controlled trials (RCTs) submitted by the manufacturer for benefit assessments resulting in conditional and unconditional appraisals in oncology, the following parameters were analysed: number of RCTs, number of patients in the largest trial, number of control arms, use of an active control (extracted from the respective manufacturer’s dossier [8]), potential for bias (extracted either from the respective IQWiG benefit assessment or the benefit decision rationale of the G-BA [8]), availability of direct comparison to appropriate comparator (extracted from the respective benefit decision rationale of the G-BA [8]).

### Categories of missing data

Data requests by the EMA and the G-BA were categorised as efficacy, safety, effectiveness, pharmacology or reference to EMA. A conditional appraisal or PAM could be included in more than one category, but each category was only counted once per appraisal or per product with PAMs. Categories were defined as follows:

- **Efficacy:** efficacy data or specific efficacy endpoint(s) explicitly mentioned in the EPAR or the G-BA decision rationale and/or request listed in the ‘conclusions on clinical efficacy’ section of the EPAR; data from (randomised) studies
- **Safety:** safety data or specific safety endpoint(s) explicitly mentioned in the EPAR or the G-BA decision rationale and/or request listed in the ‘conclusions on clinical safety’ section of the EPAR; data from safety studies (randomised or observational studies or registries)
- **Effectiveness:** data from non-interventional, observational or 'real-life' studies or registries other than for the purposes of safety monitoring, or requests for data on representative patient populations covering the entire indication
- **Pharmacology:** data on drug-drug interactions, pharmacokinetics, pharmacodynamics, dosing or dose timing
- **Reference to EMA** (only applicable for G-BA requests): Data requests consistent with PAM(s)

### Types of data requested

This assessment comprised the clarity of the request and, for clear requests, the type of data requested. A request was considered to be clear if the type of study required to obtain the requested data was unambiguously specified and either detailed guidance on e.g. endpoints was provided or protocol approval was required by the CHMP prior to study start. Requests where these details were missing were classified as unclear. Clarity was rated for each individual PAM, but only once for each conditional appraisal as a whole.

Types of data requests were categorised as follows:

- **RCT:** data from RCTs
- **Non-RCT:** data from non-randomised studies
- **Analysis:** new analyses of existing data or ongoing studies
- **Other:** e.g. simulations or in vitro studies
- **Reference to EMA** (only applicable for G-BA requests): data requests consistent with PAMs

Studies requested in the non-RCT category were further subdivided into pharmacokinetic/drug interaction studies, post-authorisation safety studies, single-arm studies, long-term studies, registries, cohort studies or other, according to the study description.

### Main topics in G-BA data requests beyond EMA data requirements

For conditional appraisals with G-BA data requests in terms of efficacy, safety and effectiveness going beyond EMA data requirements, the main topics of the requests were identified and categorised as described below. Individual requests could include more than one topic.

- **Endpoint:** Data on specific endpoints either not presented by manufacturer or not accepted by the G-BA
- **Comparator:** (Direct) comparison to the appropriate G-BA-specified comparator missing
- **Long-term data:** Data on long-term outcomes missing
- **Patient number:** Number of patients for a specific population too small
- **Population:** Further subdivision of population requested
- **Post-marketing safety concerns** (specific to safety requests): Safety concerns due to safety signals from post-marketing experience
- **Incomplete population** (specific to effectiveness requests): Study population not reflecting the full indication
- **Population not representative** (specific to effectiveness requests): Study population not comparable to patients treated in the German context

### Data extraction procedure

All assessments were conducted by two independent reviewers to increase the level of objectivity in this study. Discrepancies between reviewers were resolved through discussion.

## Results

### Analysis set

**Fig. 1:**
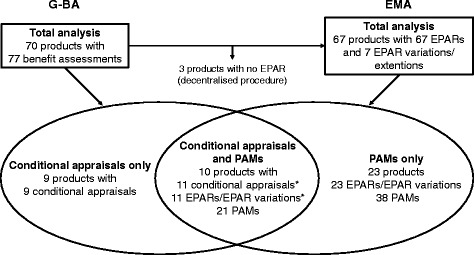
Dataset used for the analysis of conditional appraisals and PAMs * Ipilimumab received a license expansion that resulted in an EPAR variation and a second benefit assessment. EMA: European Medicines Agency; EPAR: European Public Assessment Report; G-BA: Federal Joint Committee; PAM: post-authorisation measure

The analysis included 77 early benefit assessments conducted during the 3.5-years assessment period and their corresponding EPARs/EPAR variations where available. An overview of the analysis set is shown in Fig. 1, and a complete list of products with conditional appraisals and/or PAMs can be found in Table 1.

**Table 1:** List of products with conditional appraisals^a^ and/or PAMs^b^

Product	Brand name	Indication	Conditional appraisals^d^		PAMs^e^	
			y/n	Time frame (years)	Number	Time frame (years)
Aclidinium bromide	Eklira Genuair/Bretaris Genuair	COPD	No	-	1	n.a.
Afatinib	Giotrif	Non-small-cell lung carcinoma	Yes	1	0	-
Aflibercept^f^	Zaltrap	Metastatic colorectal cancer	No	-	1	4
Aflibercept^f^	Eylea	Age-related macular degeneration	No	-	1	5
Aliskiren/amlopidine	Rasilamlo	Essential hypertension	No	-	2	1; n.a.
Axitinib	Inlyta	Renal cell carcinoma	Yes	4	0	-
Belatacept	Nulojix	Renal transplantation	Yes	3	0	-
Belimumab	Benlysta	Systemic lupus erythematodes	No	-	3	1.5; 8.5; 11.5
Boceprevir	Victrelis	Chronic hepatitis C	No	-	1	4.5
Bosutinib^g,c^	Bosulif	Chronic myeloid leukaemia	Yes	5	2	1; 5.5
Brentuximab vedotin^g,c^	Adcetris	Hodgkin lymphoma, anaplastic large-cell lymphoma	No	-	4	Annually; 3.5; 3.5; 6
Crizotinib^c^	Xalkori	Non-small-cell lung carcinoma	Yes	2	2	1.5; 3.5
Dabrafenib	Tafinlar	Melanoma	Yes	3.5	0	-
Elvitegravir/cobicistat/emtricitabine/tenofovir disoproxil	Stribild	HIV infection	No	-	1	0.5
Emtricitabine/rilpivirine/tenofovir disoproxil^h^	Eviplera	HIV infection	No	-	2	1; 2
Eribulin	Halaven	Breast cancer	Yes	2	0	-
Extract of C*annabis sativa*	Sativex	Multiple sclerosis	Yes	3	No centralised procedure	
Fampridine^c^	Fampyra	Multiple sclerosis	No	-	1	5.5
Fidaxomicin	Dificlir	Infection with clostridium	No	-	2	1.5; 1.5
Fingolimod	Gilenya	Multiple sclerosis	Yes	3	1	9.5
Fluticasone furoate/vilanterol trifenatate	Relvar Ellipta	Asthma, COPD	No	-	2	2; 2.5
Indacaterol/glycopyrronium	Ultibro Breezhaler, Xoterna Breezhaler	COPD	No	-	1	5
Ipilimumab^i^	Yervoy	Melanoma (pre-treated)	Yes	5	1	6
Ipilimumab (new indication)^i^	Yervoy	Melanoma (treatment-naïve)	Yes	3.5	1	6
Ivacaftor^g^	Kalydeco	Cystic fibrosis	No	-	2	3.5; 5.5
Lixisenatide	Lyxumia	Diabetes mellitus type 2	No	-	1	1.5
Lomitapide^c^	Lojuxta	Hypercholesterolaemia	Yes	1	2	Annually; 6.5
Ocriplasmin	Jetrea	Vitreomacular traction	Yes	5	0	-
Pertuzumab	Perjeta	Breast cancer	Yes	5	2	3.5; 4.5
Pirfenidone^g^	Esbriet	Idiopathic pulmonary fibrosis	No	-	1	6.5
Pixantrone^c^	Pixuvri	Non-Hodgkin lymphoma	No	-	1	3
Pomalidomide^g^	Imnovid	Multiple myeloma	No	-	2	1; 7
Ponatinib^g^	Iclusig	Lymphoblastic leukaemia, myeloid leukaemia	Yes	1	0	-
Regorafenib	Stivarga	Colorectal cancer	Yes	1.5	5	0.2; 0.2; 1; 2; 7.5
Rilpivirine^h^	Edurant	HIV infection	No	-	2	1; 2
Ruxolitinib^g^	Jakavi	Chronic myeloproliferative disorders	No	-	2	Annually; 1
Saxagliptin/metformin	Komboglyze	Diabetes mellitus type 2	Yes	2	0	-
Tafamidis meglumine^g,c^	Vyndaqel	Amyloidosis	No	-	1	annually
Ticagrelor	Brilique	Acute coronary syndrome	No	-	1	2.5
Trastuzumab emtansine	Kadcyla	Breast cancer	No	-	3	1; 3; 3.5
Vandetanib^j,c^	Caprelsa	Thyroid neoplasms	Yes	3	2	2; 4
Vemurafenib	Zelboraf	Melanoma	Yes	1	1	2
Vismodegib^c^	Erivedge	Basal cell carcinoma	Yes	2	2	1; 2

*COPD* chronic obstructive pulmonary disease, *n.a.* not available, *EPAR* European Public Assessment Report, *PAM* post-authorisation measure

^a^ *N* = 19 (20 appraisals);

^b^ *N* = 33 (34 EPARs);

^c^indicates conditional marketing authorisation (bosutinib, brentuximab vedotin, crizotinib, fampridine, pixantrone, vandetanib and vismodegib) or marketing authorisation under exceptional circumstances (lomitapide, tafamidis meglumine)

^d^Conditional appraisals are issued with a single time frame

^e^More than one PAM per product is possible; time frames are issued per PAM

^f^Aflibercept is marketed as Zaltrap for colorectal cancer and as Eylea for age-related macular degeneration (AMD) and central retinal vein occlusion (CRVO). Therefore, 3 benefit assessments (one for each indication) and 2 EPARs (for Eylea and Zaltrap) are available for aflibercept. The PAM given in the Eylea EPAR refers to AMD; Eylea for CRVO was therefore omitted from the table

^g^Medicinal products with orphan status

^h^For emtricitabine/rilpivirine/tenofovir disoproxil and rilpivirine, the same PAM was requested and was counted twice

^i^Ipilimumab received a license expansion that resulted in an EPAR variation and a second benefit assessment. It had one PAM that was considered applicable to both indications and was therefore counted twice

^j^In case of no additional benefit due to missing data, the manufacturer can apply for re-assessment and subsequently submit the missing data. This was done for vandetanib, and only the second assessment (with conditional appraisal) was included

The 77 benefit assessments covered 70 products as 7 products were assessed twice by the G-BA due to authorisation for a new indication. Twenty appraisals (26 %), covering 19 products, were conditional; for ipilimumab, two conditional appraisals for two different indications were included.

For 3 of the 70 products (extract of *Cannabis sativa*, lisdexamfetamine dimesylate and pitavastatin), no EPAR was available as they underwent a decentralised procedure. Therefore, 67 products with 74 EPARs/EPAR variations were included in the PAM analysis. Of these, 33 products (49 %) had 34 EPARs with at least one PAM. Seven and 2 of the 33 products had obtained conditional approval and approval under exceptional circumstances, respectively. The total number of PAMs was 59, with 30 EPARs containing only one or 2 PAMs (Table 1).

### Therapeutic indications

An analysis of the therapeutic indications of products with conditional appraisals (*N* = 19) and PAMs (*N* = 33) revealed that oncology drugs comprised less than half of the products (*n* = 14) which had a PAM, but more than two-thirds of the products (*n* = 13) for which a conditional appraisal had been issued.

### Quality of data from RCTs for oncology drugs

As a disproportionally high proportion of conditional appraisals concerned oncology drugs as compared with other indications, the trial design of RCTs presented by the manufacturer was compared for the products with conditional (*N* = 12) vs. unconditional appraisals (*N* = 9) in oncology (Table 2). In general, no distinctive differences were evident, except that RCTs in conditional vs. unconditional appraisals were more frequently considered to have a high potential for bias (67 % vs. 11 %) and were more frequently active controlled (67 % vs. 33 %).

**Table 2:** Data quality from RCTs for oncology drugs with conditional vs. unconditional appraisals^a^

	Conditional appraisals (*N* = 12)	Unconditional appraisals (*N* = 9)	Total (*N* = 21)
Number of RCTs presented in manufacturer’s dossier (mean ± SD)	1.3 ± 1.2	1.0 ± 0.5	1.2 ± 1.0
Number of patients in largest RCT (mean ± SD)	577 ± 207	939 ± 361	729 ± 329
Number of control arms (mean ± SD)	1.1 ± 0.3	1.0 ± 0	1.1 ± 0.2
Use of an active control (*n*, %)	8 (67 %)	3 (33 %)	11 (52 %)
Benefit outcome influenced by potential for bias	8 (67 %)	1 (11 %)	9 (43 %)
Direct comparison to appropriate comparator available	9 (75 %)	7 (78 %)	16 (76 %)

*RCT* randomised controlled trial, *SD* standard deviation

^a^Excluding orphan drugs

### Categories of missing data

A total of 35 and 54 data categories were assigned to the conditional appraisals and PAMs, respectively (Table 3). In line with EMA guidelines [2], main categories requested by the EMA were efficacy and safety (22/54 for both). Seven of 35 G-BA data requests were consistent with requests in the corresponding PAMs. The remaining 28 of 35 G-BA requests comprised additional data on efficacy (*n* = 13), safety (*n* = 10) or effectiveness (*n* = 5), i.e. 80 % of the G-BA data requirements went beyond requests made by the EMA. In contrast, the G-BA did not request pharmacology data, which was covered by 8 PAMs.

**Table 3:** Categories of missing data for conditional appraisals^a^ and PAMs

	Conditional appraisals (*N* = 35 categories) *n* ^b^	PAMs (*N* = 54 categories) *n* ^b^
Category		
Efficacy	13	22
Safety	10	22
Effectiveness	5	2
Pharmacology	0	8
Reference to EMA^c^	7	–

*PAM* post-authorisation measure

^a^19 products with 20 conditional appraisals, 33 products with 34 EPARs

^b^Number of data requests for each category. More than one category of data request was permissible for each PAM or conditional appraisal, but each category was only counted once for each PAM or conditional appraisal

^c^Reference to EMA only applicable for G-BA restrictions. All other G-BA data requests (efficacy, safety, effectiveness and pharmacology) were unique to the respective appraisals and were not reflected by EMA requests

### Types of data requested

**Fig. 2:**
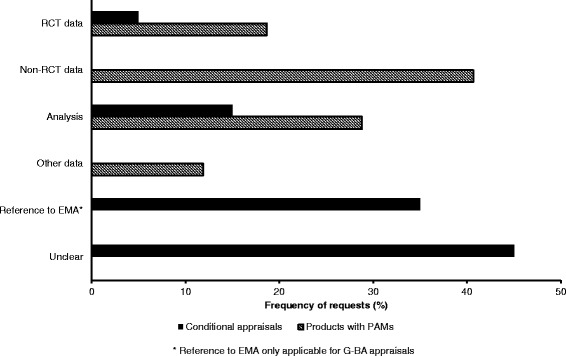
Data type requests by the G-BA^a^ and the EMA^b^ EMA: European Medicines Agency; G-BA: Federal Joint Committee; PAM: post-authorisation measure; RCT: randomised controlled trial; ^a^ per appraisal (*N* = 20); ^b^ per PAM (*N* = 59)

Data requests were initially categorised as clear or unclear in terms of the requested study design, endpoints, etc. Nine of 20 conditional appraisals but none of the 59 PAMs were considered unclear. Clear requests were further divided by data type (Fig. 2). The EMA requested the generation of new non-RCT data and new analyses of existing data or already initiated studies in 24 and 17 of 59 PAMs, respectively. Requests for non-RCT data comprised mostly pharmacokinetics/drug interaction studies (*n* = 7), post-authorisation safety studies (*n* = 5) and single-arm studies (*n* = 4) (see Table 4 for a complete list).

**Table 4:** Non-RCT data required by the EMA as PAMs (*N* = 24)

Type of non-RCT PAM	*n*
Drug interaction and PK studies	7
Post-authorisation safety studies	5
Single-arm studies	4
Long-term observational or non-interventional studies	3
Registries	2
Cohort studies	2
Other	1

*EMA* European Medicines Agency, *PAM* post-authorisation measure, *PK* pharmacokinetics, *RCT* randomised controlled trial

Of the 11/20 conditional appraisals classified as clear, most (7/20) were consistent with the respective PAMs. The remaining requests concerned new analyses and new RCT data (3/20 and 1/20, respectively). No non-RCT data were requested in accordance with the G-BA Rules of Procedure, which stipulate that RCT data are preferred for benefit assessments [5].

### G-BA data requests beyond EMA data requirements

Assessment of a product’s efficacy and safety profile typically falls into the domain of the licensing authorities, e.g. the EMA or the German national licensing authorities. As a substantial number (*n* = 14) of conditional appraisals contained requests for efficacy and safety data going beyond those made by the EMA, these were further analysed to identify the main topics covered.

A summary of this analysis is presented in Table 5. Key topics in efficacy (*N* = 18) and safety (*N* = 13) requests were endpoints that were missing or not accepted by G-BA (*n* = 8 and *n* = 2, respectively), missing data against the appropriate comparator (*n* = 4 and *n* = 5, respectively) and missing long-term data (*n* = 4 and *n* = 2, respectively). Post-marketing safety concerns accounted for 2/13 safety requests; only a minority of efficacy and safety requests concerned insufficient patient numbers (*n* = 1 for both) and inappropriate study populations (*n* = 1 for both). All effectiveness requests (*N* = 5) focused on study populations, which either did not comprise all patients included in the indication (*n* = 3) or were not considered representative for Germany (*n* = 2).

**Table 5:** Main topics of G-BA data requests beyond EMA data requirements^a^

Efficacy	Safety	Effectiveness
Endpoint [8]	Comparator [5]	Incomplete population [3]
Comparator [4]	Endpoint [2]	Population not representative [2]
Long-term data [4]	Long-term data [2]	
Patient number [1]	Post-marketing safety concerns [2]	
Population [1]	Patient number [1]	
	Population [1]	

*EMA* European Medicines Agency, *G-BA* Federal Joint Committee

Numbers in brackets indicate the number of conditional appraisals concerned; more than one topic per category was possible for each appraisal

^a^Per appraisal (*N* = 14)

## Discussion

In this analysis, we compared post-authorisation constraints from the EMA and the G-BA during the first 3.5 years after the introduction of early benefit assessment in Germany.

A considerable number of data requests by the G-BA went beyond those issued by the EMA (80 %), which may appear surprising considering a court ruling from the Federal Social Court stipulating that the G-BA may not issue a judgment on the quality, efficacy or safety of a drug that deviates from that of the licensing authorities [10]. However, according to the G-BA, the therapeutic benefit required for a drug to be reimbursable by the German sick funds is not identical to the therapeutic efficacy and the favourable benefit-risk ratio proven by the marketing authorisation [11]. In line with this, the Federal Social Court acknowledged that there may be discrepancies between the evidence gathered from pivotal trials and the requirements concerning the use of a drug in clinical practice [12].

In particular, the G-BA considers itself not bound by decisions made by the licensing authorities regarding appropriate comparator and patient-relevant endpoints [13], two areas of special relevance in the determination of additional benefit. Indeed, the majority of additional requests for both efficacy and safety data specifically concern these two topics, which have previously been identified as aspects in which G-BA requirements substantially deviate from the EMA’s viewpoint [7, 14–16]. A recent evaluation of parallel scientific advice meetings demonstrated that the level of disagreement between the EMA and health technology assessment (HTA) bodies regarding the comparator and endpoints was 30 % and 12 %, respectively [17], indicating that the different remits and perspectives of regulators and HTA bodies frequently cause discrepancies regarding these points.

While the practice of issuing conditional appraisals and requesting additional data has a clear legal basis and is not questioned *per se*, this analysis identifies key shortcomings in the current G-BA approach such as a lack of rationale for conditional appraisals, a lack of methodological guidance from the G-BA and a lack of flexibility and pragmatism within the G-BA.

Our results demonstrate that the reasons for issuing conditional appraisals are not transparent as the quality of the submitted data for oncology assessments was overall comparable between conditional vs. unconditional appraisals, except for minor deviations in the potential for bias. In line with this, an analysis of G-BA appraisals concerning products approved under special regulatory circumstances showed that clear reasons for issuing conditional or unconditional appraisals were not always reported in the documentation of the assessment procedure [18].

Data requests by the G-BA are issued without any methodological guidance to assist the manufacturer in generating acceptable data [8]. In contrast, the majority of PAMs requesting additional studies stipulated that the study protocol should be approved by the CHMP prior to study start [9]. This ensures alignment between the manufacturer and the EMA and enables the manufacturer to adequately address PAM requests.

Other European HTA bodies also provide guidance regarding their preferred methodology. The French authority, the Haute Autorité de Santé, can request additional data, which may require new studies, during its assessment procedure. In order to make sure the agency’s requirements are met, a guidance document regarding general methodological considerations has been published, and the study protocol has to be submitted for evaluation prior to the start of the study [19].

The re-assessment of benefit after the expiry of the conditional appraisal for vemurafenib illustrates the consequences of the absence of such guidance by the G-BA. In its initial assessment, the G-BA had suggested a historic comparison of dacarbazine vs. vemurafenib to allow an evaluation of the differences in overall survival. Although this was provided by the manufacturer in a revised dossier, the G-BA concluded that it was obsolete for methodological reasons and the initially assigned additional benefit rating was not changed [8].

Our analysis reveals obvious differences in data handling between G-BA and EMA, with the EMA showing considerably more flexibility in terms of data acceptance, which may explain why almost half of the products with a conditional appraisal received no PAM. Of note, the EMA frequently had similar concerns to the G-BA, but took additional data into account to answer open questions. This is partly due to the consideration of studies involving different comparators and studies in which the therapy under assessment was not administered at the approved dose; most importantly, however, the EMA is not limited to RCTs in its evaluations. Whereas the G-BA mainly follows the methodology laid down by the IQWiG, which requests RCT data wherever feasible [20], the EMA also uses sources such as non-clinical studies or even expert advice to put data from pivotal trials into context.

A similarly less restrictive approach explicitly accepting non-RCT data is used by the UK National Institute for Health and Clinical Excellence (NICE) for HTA [21]; in such cases, data adjustment by modelling and a critical appraisal of uncertainty are required. Modelling is generally used to extrapolate data that do not fit ideal requirements, e.g. surrogate endpoints, a shorter than desired study duration or a study population that is not representative of patients within the UK National Health Service [22]. NICE therefore tries to make the most of the available data and to obtain at least limited information, whereas the G-BA in most cases categorically rejects all data that do not conform to the IQWiG’s standards and does not take this information into account. As the vemurafenib example shows, the rigid data requirements of the G-BA, together with the lack of methodological guidance, also represent a significant obstacle to obtaining a superior benefit rating in a re-assessment, even if new data are submitted.

For medicines with conditional appraisals, as the initially presented data have not been considered to be fully conclusive by the G-BA, it can be expected that the benefit rating is conservatively low. This will influence initial reimbursement as under AMNOG, the price of a medicine is in part determined by its benefit rating [23]. In contrast, an improved benefit rating after re-assessment would result in new price negotiations and potentially in a higher price. Considering that the goal of AMNOG is to reduce the rapidly increasing drug costs to the statutory health insurance by 2.2 billion Euro per year [24], it could be hypothesised that this may not be the desired outcome in the eyes of the G-BA and the German sick funds. In fact, a potential political motivation regarding data evaluation, namely in the determination of the appropriate comparator by the G-BA, in order to contain costs has been suspected before [25].

In a worst case scenario, a drug can actually receive a worse benefit rating after re-assessment, which would very likely further lower its price. This occurred in the case of regorafenib [8], which was consequently taken off the German market after the re-assessment because the manufacturer was not willing to further reduce the price [26]. It has already been noted that AMNOG has decreased the traditionally high availability of innovative drugs on the German market through opt-outs following benefit assessments [27]; conditional appraisals and the resulting re-assessments may well contribute to this.

A structured collaboration between the German national licensing authorities and the G-BA, in the form of national joint scientific advice, has recently been established [28]. While this is a positive development, it has already been clarified that this dialogue “will not, cannot and is not intended to” harmonise study requirements [29]. This will continue to make it difficult for manufacturers to obtain data acceptable to both the EMA and the G-BA in one single study. The previously cited analysis of joint scientific advice meetings [17] showed that in cases where the EMA and HTA bodies did not agree on the comparator, the suggested solution was most frequently an indirect comparison. However, given the stringent methodological requirements in Germany, it will be challenging to obtain an additional benefit on this basis: of the first 23 indirect comparisons submitted after the introduction of AMNOG, only one was formally accepted by the IQWiG [25].

A genuine harmonisation of study requirements between regulators and HTA bodies has repeatedly been called for, not just in relation to the G-BA [30, 31] but for European HTA in general [17, 32]. More flexibility in HTA will be especially crucial as new methods for marketing authorisation emerge [33]. Alignment of data requests between the stakeholders, both around the time of authorisation and during a product’s life cycle, would markedly simplify the process of data generation to answer important questions concerning a given drug.

This analysis has some limitations. Firstly, when reviewing the Procedural Steps, only PAMs clearly identified as such were included. Therefore, studies originally conducted as PAMs but not appropriately labelled may have been excluded from the analysis. However, as only products with newly granted marketing authorisations were assessed, it can be assumed that the majority of PAMs have not yet been fulfilled, and therefore the number of PAMs not included is probably negligible. Secondly, the dataset is relatively small as only appraisals from the first 3.5 years of AMNOG were analysed and the number of available benefit assessments from this period is limited. Future studies on larger datasets will be required to validate the conclusions drawn from this analysis.

## Conclusions

In conclusion, it would be helpful if the factual basis for conditional appraisals could be clarified. Moreover, clear instructions regarding the fulfilment of data requests, as typically provided for PAMs, would be beneficial in providing direction to the manufacturer and subsequently enabling the G-BA to make an informed decision on the additional benefit of a new medicinal product. Ideally, discussions between the licensing authorities, the G-BA and manufacturers early in a product’s life cycle should determine the extent and format of the required data and the strategies to be used for their generation. Undoubtedly, everybody, from licensing authorities over HTA bodies and payers to clinicians and patients would stand to benefit from this scenario.

## Abbreviations

AMNOG: Act for Restructuring the Pharmaceutical Market in Statutory Health Insurance
CHMP: Committee for Medicinal Products for Human Use
EMA: European Medicines Agency
EPAR: European Public Assessment Report
FDA: U.S. Food and Drug Administration
G-BA: German Federal Joint Committee
HTA: Health technology assessment
IQWiG: Institute for Quality and Efficiency in Health Care
NICE: National Institute for Health and Clinical Excellence
PAM: Post-authorisation measure
RCT: Randomised controlled trial

## References

[1] Food and Drug Administration. Postmarketing Studies and Clinical Trials - Implementation of section 505(o)(3) of the Federal Court Food, Drug and Cosmetic Act. 2011. http://www.fda.gov/downloads/Drugs/GuidanceComplianceRegulatoryInformation/Guidances/UCM172001.pdf. Accessed 18 Aug 2016.

[2] European Medicines Agency. European Medicines Agency post-authorisation procedural advice for users of the centralised procedure. 2014. http://www.ema.europa.eu/docs/en_GB/document_library/Regulatory_and_procedural_guideline/2009/10/WC500003981.pdf. Accessed 18 Aug 2016.

[3] Bundesministerium für Gesundheit. [AM-NutzenV]. 2010. http://www.gesetze-im-internet.de/bundesrecht/am-nutzenv/gesamt.pdf. Accessed 18 Aug 2016.

[4] Deutscher Bundestag. [Sozialgesetzbuch (SGB) Fünftes Buch (V) - Gesetzliche Krankenversicherung - (Artikel 1 des Gesetzes v. 20. Dezember 1988, BGBl. I S. 2477, letzte Änderung durch Artikel 3 des Gesetzes vom 20. Dezember 2012 (BGBI. I S. 2781))]. 1988. http://www.gesetze-im-internet.de/bundesrecht/sgb_5/gesamt.pdf. Accessed 18 Aug 2016.

[5] Gemeinsamer Bundesausschuss. Chapter 5: Assessment of the benefits of pharmaceuticals according to §35a SGB V. 2012. http://www.english.g-ba.de/downloads/17-98-3042/2011-05-18_5%20Kapitel%20VerfO%20Englisch.pdf. Accessed 18 Aug 2016

[6] Hörn H, Nink K, McGauran N, Wieseler B (2014). Early benefit assessment of new drugs in Germany - Results from 2011 to 2012. Health Policy.

[7] Ruof J, Schwartz FW, Schulenburg JM, Dintsios CM (2014). Early benefit assessment (EBA) in Germany: analysing decisions 18 months after introducing the new AMNOG legislation. Eur J Health Econ.

[8] Gemeinsamer Bundesausschuss. Overview of products [Übersicht der Wirkstoffe]. 2016. http://www.g-ba.de/informationen/nutzenbewertung/. Accessed 18 Aug 2016

[9] European Medicines Agency. European public assessment reports. 2016. http://www.ema.europa.eu/ema/index.jsp?curl=pages/medicines/landing/epar_search.jsp&mid=WC0b01ac058001d124. Accessed 18 Aug 2016.

[10] Bundessozialgericht. [Urt. v. 31.05.2006, Az.: B 6 KA 13/05 R]. 2006. http://www.aok-business.de/fachthemen/pro-personalrecht-online/datenbank/urteile-ansicht/poc/docid/2505916/. Accessed 18 Aug 2016.

[11] Gemeinsamer Bundesausschuss. [Stellungnahme des G-BA zum Fraktionsentwurf von CDU/CSU und FDP zu einem Gesetz zur Neuordnung des Arzneimittelmarktes in der gesetzlichen Krankenversicherung (Arzneimittelmarktneuordnungsgesetz - AMNOG; BT/Drucks. 17/2413) - Korrigierte, um Punkt 8. auf Seite 7 erweiterte Fassung -]. 2010. https://www.g-ba.de/downloads/17-98-2896/Stellungnahme%20G-BA%20zum%20AMNOG_Anh%C3%B6rung%2029-09-2010.pdf. Accessed 18 Aug 2016.

[12] Bundessozialgericht. [Urt. v. 01.03.2011, Az.: B 1 KR 7/10 R]. 2011. https://openjur.de/u/169319.html. Accessed 18 Aug 2016.

[13] Grüne M. [„Bedeutung der Zulassung für die Nutzenbewertung“- Stellenwert des Zulassungsstatus bei G-BA- Entscheidungen]. 2013. https://www.iqwig.de/download/13-06-21_IQWiG_im_Dialog_Maximilian_Gruene_Bedeutung_der_Zulassung_Nutzenbewertung_Stellenwert_des_Zulassungsstatus_bei_G-BA-Entscheidungen.pdf. Accessed 18 Aug 2016.

[14] Dabisch I, Dethling J, Dintsios CM, Drechsler M, Kalanovic D, Kaskel P (2014). Patient relevant endpoints in oncology: current issues in the context of early benefit assessment in Germany. Health Econ Rev.

[15] Kvitkina T, ten Haaf A, Reken S, McGauran N, Wieseler B (2014). Patient-relevant outcomes and surrogates in the early benefitassessment of drugs: first experiences [Patientenrelevante Endpunkte und Surrogate in der frühen Nutzenbewertung von Arzneimitteln: erste Erfahrungen]. Z Evid Fortbild Qual Gesundhwesen.

[16] Ruof J, Knoerzer D, Duenne AA, Dintsios CM, Staab T, Schwartz FW (2014). Analysis of Endpoints Used in Marketing Authorisations versus Value Assessments of Oncology Medicines in Germany. Health Policy.

[17] Tafuri G, Pagnini M, Moseley J, Massari M, Petavy F, Behring A (2016). How aligned are the perspectives of EU regulators and HTA bodies? A comparative analysis of regulatory-HTA parallel scientific advice. Br J Clin Pharmacol.

[18] Ecker T, Fink C, Puetz C. Assessing medicinal products with limited evidence in Germany – current experience. Value Health. 2015;18(7):A551. (abstract and poster PHP213 presented at ISPOR 2015, Milan, Italy).

[19] Haute Autorité de Santé. [Les études post-inscription sur les technologies de santé (médicaments, dispositifs médicaux et actes) - Principes et méthodes]. 2011. http://www.has-sante.fr/portail/upload/docs/application/pdf/2012-01/etudes_post_inscription_technologies_sante.pdf. Accessed 18 Aug 2016. 10.2515/therapie/2012065 27393714

[20] Institut für Qualität und Wirtschaftlichkeit im Gesundheitswesen. General Methods 4.2. 2015. https://www.iqwig.de/download/IQWiG_General_Methods_Version_%204-2.pdf. Accessed 18 Aug 2016.

[21] Ivandic V (2014). Requirements for benefit assessment in Germany and England - overview and comparison. Health Econ Rev.

[22] National Institute for Health and Clinical Excellence. Guide to the methods of technology appraisal 2013. 2013. https://www.nice.org.uk/process/pmg9/chapter/1-foreword. Accessed 18 Aug 2016. 27905712

[23] [Rahmenvereinbarung nach § 130b Abs. 9 SGB V]. 2015. https://www.gkv-spitzenverband.de/media/dokumente/krankenversicherung_1/arzneimittel/rahmenvertraege/pharmazeutische_unternehmer/Arzneimittel_RV_nach_130b_Abs_9_SGB-V_20150826.pdf. Accessed 18 Aug 2016.

[24] Bundesministerium für Gesundheit. [Das Gesetz zur Neuordnung des Arzneimittelmarktes (AMNOG)]. http://www.bmg.bund.de/krankenversicherung/arzneimittelversorgung/amnog/amnog.html. Accessed 18 Aug 2016.

[25] Lebioda A, Gasche D, Dippel FW, Theobald K, Plantor S (2014). Relevance of indirect comparisons in the German early benefit assessment and in comparison to HTA processes in England, France and Scotland. Health Econ Rev.

[26] Deutsche Gesellschaft für Hämatologie und Onkologie. [Politischer Streit auf dem Rücken der Krebspatienten – Marktrücknahme von Regorafenib]. 2016. https://www.dgho-service.de/dgho/informationen/presse/pressemitteilungen/politischer-streit-auf-dem-ruecken-der-krebspatienten-2013-marktruecknahme-von-regorafenib. Accessed 18 Aug 2016.

[27] Cassel D, Ulrich V (2015). AMNOG auf dem ökonomischen Prüfstand - Funktionsweise, Ergebnisse und Reformbedarf der Preisregulierung für neue Arzneimittel in Deutschland.

[28] Pharma Fakten. [Zulassung und Nutzenbewertung - Bundesinstitute und G-BA wollen gemeinsam Kriterien verbessern]. 2016. https://www.pharma-fakten.de/news/details/376-zulassung-und-nutzenbewertung-bundesinstitute-und-g-ba-wollen-gemeinsam-kriterien-verbessern/. Accessed 18 Aug 2016.

[29] Gemeinsamer Bundesausschuss, Bundesinstitut für Arzneimittel und Medizinprodukte, Paul-Ehrlich-Institut. Strukturierte Zusammenarbeit zwischen dem Gemeinsamen Bundesausschuss, dem Bundesinstitut für Arzneimittel und Medizinprodukte und dem Paul-Ehrlich-Institut. 2016. http://www.bmg.bund.de/fileadmin/dateien/Downloads/P/Pharmadialog/Vereinbarung_G-BA-BOB_2016-04-12.pdf. Accessed 18 Aug 2016.

[30] Arbeitsgemeinschaft der Wissenschaftlichen Medizinischen Fachgesellschaften. [Stellungnahme der Arbeitsgemeinschaft der Wissenschaftlichen Medizinischen Fachgesellschaften zum Verfahren der Frühen Nutzenbewertung von Arzneimitteln nach §35a SGBV und aufgrund des Arzneimittelmarktneuordnungsgesetzes (AMNOG) von 2010 vom 24.02.2015]. 2015. http://www.awmf.org/fileadmin/user_upload/Stellungnahmen/Medizinische_Versorgung/AWMF-Stellungnahme_AMNOG_2015-02-24.pdf. Accessed 18 Aug 2016.

[31] Leverkus F, Chuang-Stein C (2016). Implementation of AMNOG: An industry perspective. Biom J.

[32] Beinlich P, Muller-Berghaus J, Sudhop T, Vieths S, Broich K (2015). Interplay between marketing authorization and early benefit assessment of drugs. Bundesgesundheitsblatt, Gesundheitsforschung, Gesundheitsschutz.

[33] Leyens L, Brand A (2016). Early patient access to medicines: health technology assessment bodies need to catch Up with New marketing authorization methods. Public Health Genomics.

